# Intermittent Administration of Parathyroid Hormone [1–34] Prevents Particle-Induced Periprosthetic Osteolysis in a Rat Model

**DOI:** 10.1371/journal.pone.0139793

**Published:** 2015-10-06

**Authors:** Fanggang Bi, Zhongli Shi, Chenhe Zhou, An Liu, Yue Shen, Shigui Yan

**Affiliations:** Department of Orthopedic Surgery, the Second Affiliated Hospital, School of Medicine, Zhejiang University, Hangzhou, China; Nanjing Medical University, CHINA

## Abstract

We examined whether intermittent administration of parathyroid hormone [1–34] (PTH[1–34]; 60 μg/kg/day) can prevent the negative effects of titanium (Ti) particles on implant fixation and periprosthetic osteolysis in a rat model. Eighteen adult male rats (12 weeks old, bones still growing) received intramedullary Ti implants in their bilateral femurs; 6 rats from the blank group received vehicle injections, and 12 rats from the control group and PTH treatment group received Ti particle injections at the time of operation and intra-articular injections 2 and 4 weeks postoperatively. Six of the rats that received Ti particles from the PTH group also received PTH[1–34] treatment. Six weeks postoperatively, all specimens were collected for assessment by X-ray, micro-CT, biomechanical, scanning electron microscopy (SEM), and dynamic histomorphometry. A lower BMD, BV/TV, Tb.N, maximal fixation strength, and mineral apposition rate were observed in the control group compared to the blank group, demonstrating that a periprosthetic osteolysis model had been successfully established. Administration of PTH[1–34] significantly increased the bone mineral density of the distal femur, BV/TV, Tb.N, Tb.Th, Tb.Sp, Con.D, SMI, and maximal fixation strength in the PTH group compared to that in the control group. SEM revealed higher bone–implant contact, thicker lamellar bone, and larger trabecular bone area in the PTH group than in the control group. A higher mineral apposition rate was observed in the PTH group compared to both the blank and control groups. These findings imply that intermittent administration of PTH[1–34] prevents periprosthetic osteolysis by promoting bone formation. The effects of PTH[1–34] were evaluated at a suprapharmacological dosage to the human equivalent in rats; therefore, additional studies are required to demonstrate its therapeutic potential in periprosthetic osteolysis.

## Introduction

More than one million cases of total joint replacements are performed annually in the United States [1]. Although the procedures have significantly contributed to the treatment of arthritis, aseptic loosening, a major complication of the procedure, always leads to eventual failure of arthroplasty [2]. More than 40,000 cases of revision operations were performed in the United States in 1994 according to statistics [3]. Particulate wear debris from the implant surfaces, which can cause an intense inflammatory foreign-body reaction resulting in massive bone loss, is the leading cause of aseptic loosening [4, 5]. There are currently no effective pharmacological treatments to prevent bone loss, and the only option is surgical revision, which is associated with potentially destructive complications of peri-implant infection. Patients with high operative risks greatly benefit from noninvasive treatment approaches.

Many studies have demonstrated that particle-induced osteolysis and implant loosening mainly result from increased bone resorption. Macrophages and giant cells activated by particles secrete inflammatory factors that mediate bone resorption, leading to bone loss [6]. Some inflammatory cytokines, including interleukin–1 (IL–1), IL–6, tumor necrosis factor (TNF), and receptor activator of NF-κB ligand (RANKL), have been demonstrated to play important roles in the inflammatory reaction [7, 8]. Anticatabolic agents, such as cytokine antagonists, which mediate the bone resorption process, and bisphosphonates have been evaluated for the prevention or treatment of osteolysis and implant loosening [3, 9–11]. However, there is no clinical evidence that bisphosphonates have therapeutic effects on periprosthetic osteolysis [12]. With regards to other potential therapeutic agents, such as TNF antagonists [13, 14], RANKL antagonists [15], IL–6, IL–10, and IL–1 receptor antagonists [16–18], osteogenic protein 1 (OP–1) [19], erythromycin [20], α-calcitonin [21], pan-caspase [22], substance P, and hydroxymethylglutaryl-coenzyme A reductase inhibitor [23, 24], only OP–1 appears to have an anabolic effect on bone metabolism [19]. However, the authors observed that OP–1 increased the number of cancellous bone but did not significantly alter bone formation. Therefore, we focused on strategies to promote bone formation in particle-induced periprosthetic osteolysis.

Modulation of the Wnt pathway to manipulate bone remodeling is becoming a new strategy to promote bone formation [25]. Parathyroid hormone (PTH) is an anabolic factor for chondrocytes and osteocytes [26, 27]. PTH[1–34], the 1–34 amino acid segment of PTH, has been approved for the treatment of postmenopausal osteoporosis by intermittent subcutaneous injection [28]. It preserves bone formation through its survival action to osteoblasts, which is mediated by PKA and Wnt/β-catenin [29, 30]. It promotes the secretion of osteoprotegerin (OPG), an antagonist of RANK, which can block the action of osteoclasts activated by RANK [31]. Anabolic agents targeting the Wnt signaling pathway represent a promising new alternative for the prevention of periprosthetic osteolysis and aseptic loosening [32]. PTH [1–34] also promotes tendon-bone healing, repairs bone fractures, and induces periprosthetic osteointegration *in vivo* [33–36]. However, it is unknown whether PTH[1–34] can prevent periprosthetic bone loss while simultaneously stimulating bone formation of the trabecular bone around the implants.

We hypothesized that it may be useful for the treatment of particle-induced osteolysis. Our hypothesis was evaluated in a well-established experimental rat model of periprosthetic osteolysis. We found increased periprosthetic bone formation and higher push-out strength in the PTH group than in the control. These findings imply that intermittent administration of PTH[1–34] prevents periprosthetic osteolysis by promoting bone formation. This method may enable a new alternative for preventing periprosthetic osteolysis.

## Materials and Methods

### Study design

The study design and surgical protocol were reviewed and approved by the Zhejiang University Institutional Animal Care and Use Committee. In all, 18 male Sprague-Dawley rats (12 weeks old, 450 ± 20 g) were used to establish a simple and reproducible animal model for particle-induced osteolysis. The rats were divided evenly into three groups at random: blank group, control group, and PTH group (n = 6 per group). Animals from the blank and control groups received a vehicle injection, and those of the PTH group received recombinant PTH[1–34] (60 μg/kg/day, Bachem, Bubendorf, Switzerland) daily from day 1 to 6 weeks postoperatively. All rats received intramuscular injections of a fluorescence tracer (calcein; 5 mg/kg; Sigma) and tetracycline hydrochloride (25 mg/kg; Sigma) at 14 and 4 days, respectively, before euthanasia to label newly mineralized bone [37]. Six weeks after surgery, all animals were euthanized with an overdose intraperitoneal injection of pentobarbital sodium. Bilateral femurs of the hind leg were collected for analyses.

### Implants and particles

Ti_6_Al_4_V rods (1.5 mm in diameter, 10 mm in length) were provided by the Experimental Research Center of Mechanics, Zhejiang University. Commercially pure Ti particles were purchased from Alfa Aesar (mean diameter 3.0 μm, 93% < 20 μm). Particles were mixed with 95% ethanol using a magnetic stirrer for sterilization for 24 h, as described previously [38]. Endotoxin levels were ensured to be below 0.25 EU/mL by a limulus assay (chromogenic TAL endpoint assay kit, Xiamen Houshiji, CHN). Particles were resuspended in 6% rat serum in sterile phosphate buffered saline (PBS) with streptomycin (100 U/mL) and penicillin (100 U/mL) at a concentration of 1.2×10^8^/mL and stored at 4°C.

### Surgical procedure

All experimental rats underwent intramedullary implantation of the Ti rods in bilateral femurs. The surgical procedure was performed under strictly aseptic conditions. Pentobarbital sodium solution was used for anesthesia by intraperitoneal injection (Kyoritsu-seiyaku, 50 mg/kg body weight). A lateral parapatellar approach was used to expose the knee joint, and a bone canal (10 mm long, parallel to the long axis of femur) was created with a 1.5 mm diameter drill in the intercondylar fossa. In the control and PTH groups, 100 μL Ti particle suspension was injected into the canal. In the blank group, an equal volume of vehicle solution was injected. The rod was pressed into the canal immediately after administration of vehicle or Ti particle solution. The incision was closed using a 4–0 Ethibond suture. Animals were allowed to move without restriction in their cages after surgery. In the second and fourth weeks postoperatively, after anesthesia with pentobarbital sodium solution, 100 μL vehicle solution was re-injected into the knee joint cavity of animals in the blank group with a 1 mL syringe, and an equal volume of Ti particle suspension was used in the control and PTH groups.

### Radiological examination

**Fig 1:**
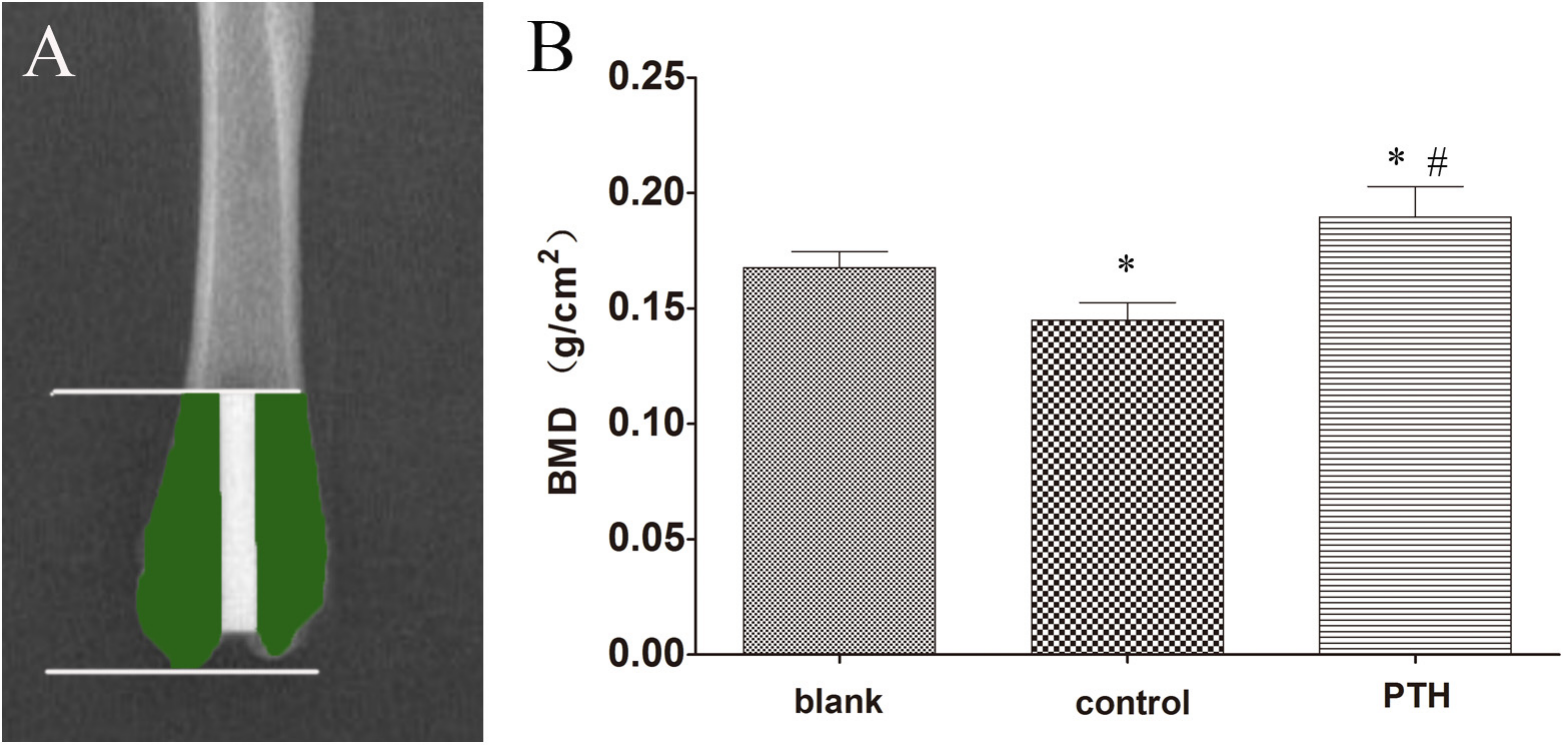
(A) The bone mineral density (BMD) around the implant of the distal femur (shaded part) was detected. (B) Histogram revealing BMDs from all three groups. *p < 0.05 vs. the blank group; #*p* < 0.05 vs. the control group.

**Fig 2:**
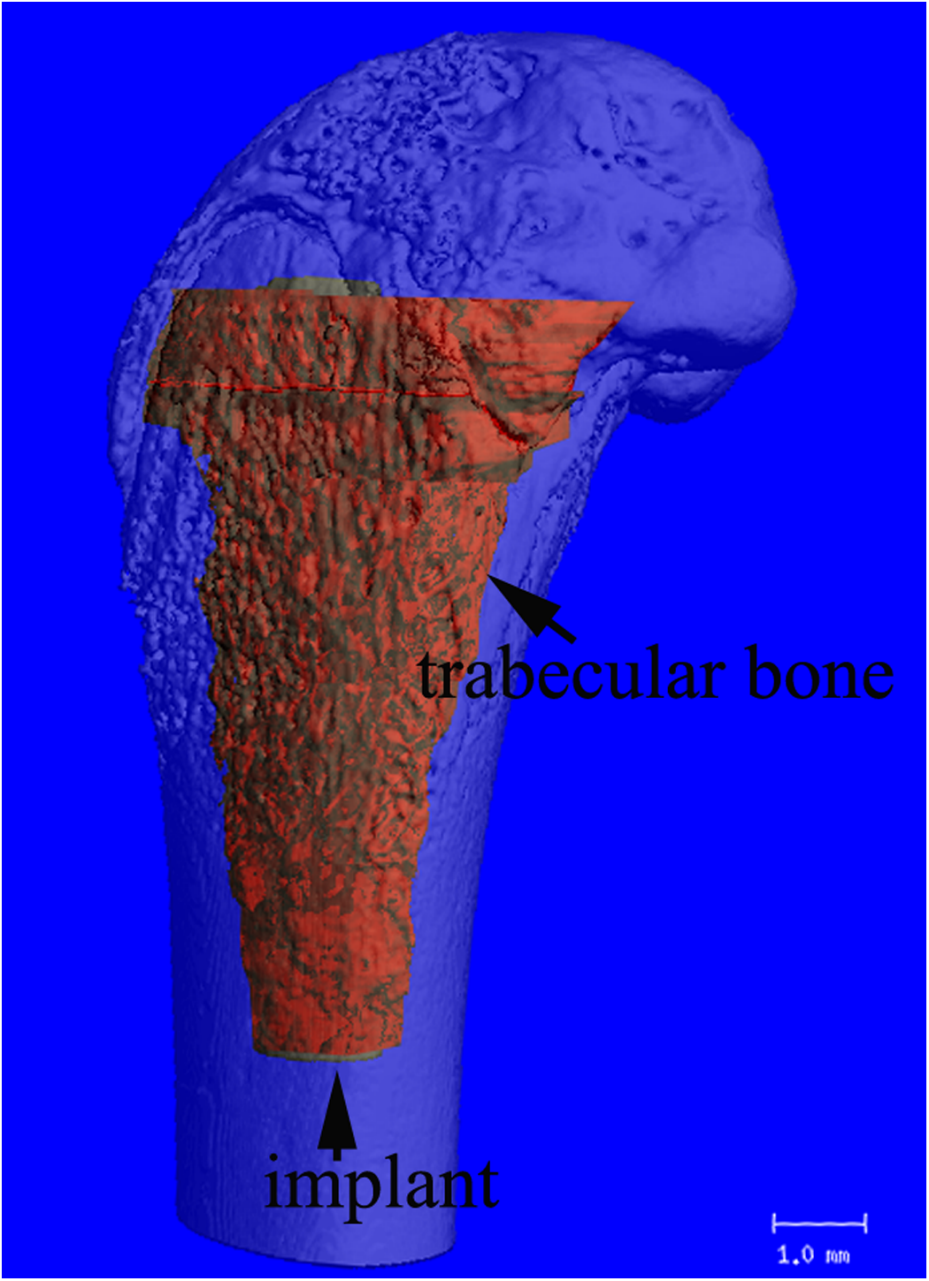
A 3D image illustrating the precise region of interest (trabecular bone between the implant and cortex, red region) evaluated by micro-CT.

Specimens were kept at -80°C immediately after collection (n = 6 per group and from different rats), and thawed at 4°C overnight before testing. Images were examined for the presence or absence of a radiolucent region around the implant using radiography at 40 kV for 5 ms. The bone mineral density (BMD) around the implant of the distal femur was detected using dual-energy X-ray absorptiometry (DEXA; GE Lunar Prodigy, Madison, WI, USA; Fig 1A). Micro-CT (20 μm thickness; Skyscan 1176, BRUKER, Antwerp, Belgium) was used to determine the trabecular bone around the rods (Fig 2). Bone volume fraction (BV/TV), bone surface/volume ratio (BS/BV), trabecular thickness (Tb.Th), trabecular number (Tb.N), trabecular separation (Tb.Sp), connective density (Conn.D), and structural model index (SMI) of the trabecular bone around the rods were determined by three-dimensional (3D) standard microstructural analysis, as described previously [33].

### Interfacial fixation strength evaluation

**Fig 3:**
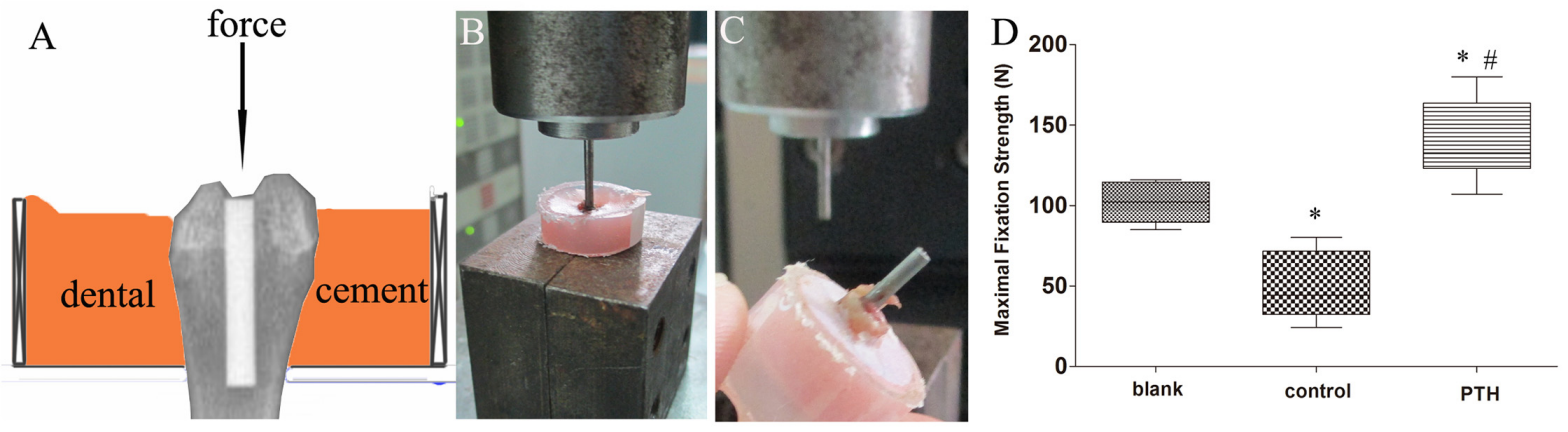
(A) Sketch map illustrating how the specimen was fixed in dental cement and the force was parallel to the long axis of the implant. (B) The specimen containing the implant was fixed in centrifugal tubes with dental cement and mounted onto the testing platform to perform the bio-mechanical tests. (C) The implant was pushed out from the specimen. (D) Statistical analyses of the bio-mechanical test data. *p < 0.05 vs. the blank group; #*p* < 0.05 vs. the control group.

The interfacial fixation strength of the specimens (n = 6 per group) was evaluated using a push-out test after a micro-CT scan. Prior to the biomechanical test, the distal femur containing the implant was vertically immobilized in hard paper with an opportune hole, allowing the distal femur to pass through. Dental cement was used to fix the specimen and a section of centrifugal tube was used to prohibit the dental cement from draining. Specimens were strictly maintained vertically to the hard paper for the force to be parallel to the long axis of the implant, allowing it to push the implant out of the bone (Fig 3A). The distal femur containing the implant was fixed in dental cement. When the cement hardened, the proximal femur was removed with a fretsaw. Specimens were mounted onto the testing platform of a Zwick/Roell 2.5 material testing system (Zwick, Ulm, Germany). A pushing load parallel to the longitudinal axis of the implant was applied at a constant speed of 1 mm/min (Fig 3B and 3C). Peak load and elongation were recorded in the load-deformation curve.

### Histomorphometric analysis

**Fig 4:**
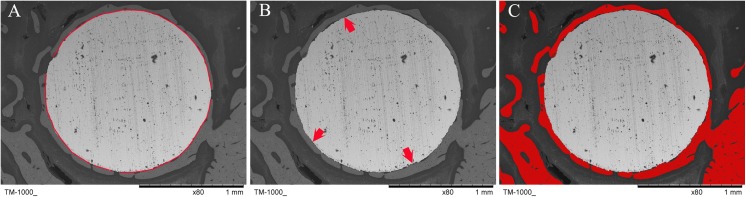
SEM image from the blank group used to illustrate how the histomorphometric analysis was performed. (**A**) Bone–implant contact: the length of contact area (red line)/perimeter of the implant; (**B**) thickness of lamellar bone (red arrows) around the implant; and (**C**) trabecular area in the SEM visual field (red area).

Bone morphogenesis around the implants was examined to assess potential bone formation following PTH[1–34] treatment. The specimens containing implants were embedded in resin for sectioning (n = 6 per group from different rats). Sections (50 μm thickness) were cut perpendicularly to the longitudinal axis of the implants using a saw microtome (Leica-SP1600, Leica Biosystems, Nussloch, Germany). Scanning electron microscope (SEM, TM–1000, Japan) images of the sections were used to assess bone–implant contact, thickness of lamellar bone around the implant, and trabecular area in the SEM visual field (Fig 4A–4C). The labeled bone surface and interlabeled thickness were detected by fluorescence microscopy (Leica DM5 500B, Leica Microsystems, Bensheim, Germany) and using Image-Pro Plus 6.0 software (IPP 6.0, Media Cybernetics Inc., Rockville, MD, USA). The data were used to calculate mineral apposition rate as follows: mineral apposition rate = interlabeled thickness per 10 days (expressed as μm/day). Then the sections were stained with a tartrate-resistant acid phosphatase (TRAP) staining kit following the manufacturer’s protocol (Sangon Biotech, Shanghai, CHN) to investigate changes at the cellular level.

### Statistical analysis

Data are expressed as the mean ± standard deviation (SD). SPSS 16.0 software (SPSS, Chicago, IL, USA) was used to analyze the data by one-way analysis of variance (ANOVA) with Scheffe’s multiple comparison method, and *p* < 0.05 was considered statistically significant.

## Results

### Radiological examination

No obvious radiolucent regions were observed in any of the specimens from the three groups. However, the BMD of the periprosthetic bone in the control group (0.145 ± 0.008 g/cm^2^) was significantly decreased compared to the blank group (0.168 ± 0.007 g/cm^2^). In the PTH group, the BMD of the same area was significantly higher than that of both the blank and control groups (*p* = 0.000 and 0.005, respectively; Fig 1B).

For micro-CT assessment, particle treatment led to a 51% reduction in BV/TV in the control group compared to the blank group (*p* < 0.05), while particle-injected rats that received PTH[1–34] daily had 4.7-fold greater BV/TV than that of the control group (*p* < 0.01). Compared to rats from the blank group, particle-injected rats that had been treated with PTH[1–34] had 2.4-fold greater BV/TV (*p* < 0.01). Table 1 describes detailed micro-CT evaluations of the periprosthetic trabecular bone and reveals similar positive results (Table 1).

**Table 1:** Micro-CT evaluations for trabecular bone between implant and cortex (mean ± SD).

Items	blank	control	PTH
BV/TV (%)	12.40±3.18	6.37±4.13*	30.00±4.95* #
BS/BV (1/mm)	37.2998±1.5359	34.9128±11.5851	24.3732±3.4829*
Tb.N (1/mm)	2.1952±0.4214	1.0941±0.4724*	3.1025±0.1496* #
Tb.Th (μm)	78.10±17.51	58.33±11.77	136.88±40.37* #
Tb.Sp (μm)	441.78±87.12	1152.68±808.68	277.37±64.88*
Conn.D (1/mm^3^)	25.0863±4.5036	16.3937±1.5047*	41.2685±1.2930* #
SMI	2.0734±0.0543	3.8024±0.2663*	1.1463±0.2154* #

*p < 0.05 vs. the blank group

#*p* < 0.05 vs. the control group. BV/TV, bone volume fraction; BS/BV, bone surface/volume ratio; Tb.N, trabecular number; Tb.Th, trabecular thickness; Tb.Sp, trabecular separation; Conn.D, connective density; SMI, structural model index.

**Fig 5:**
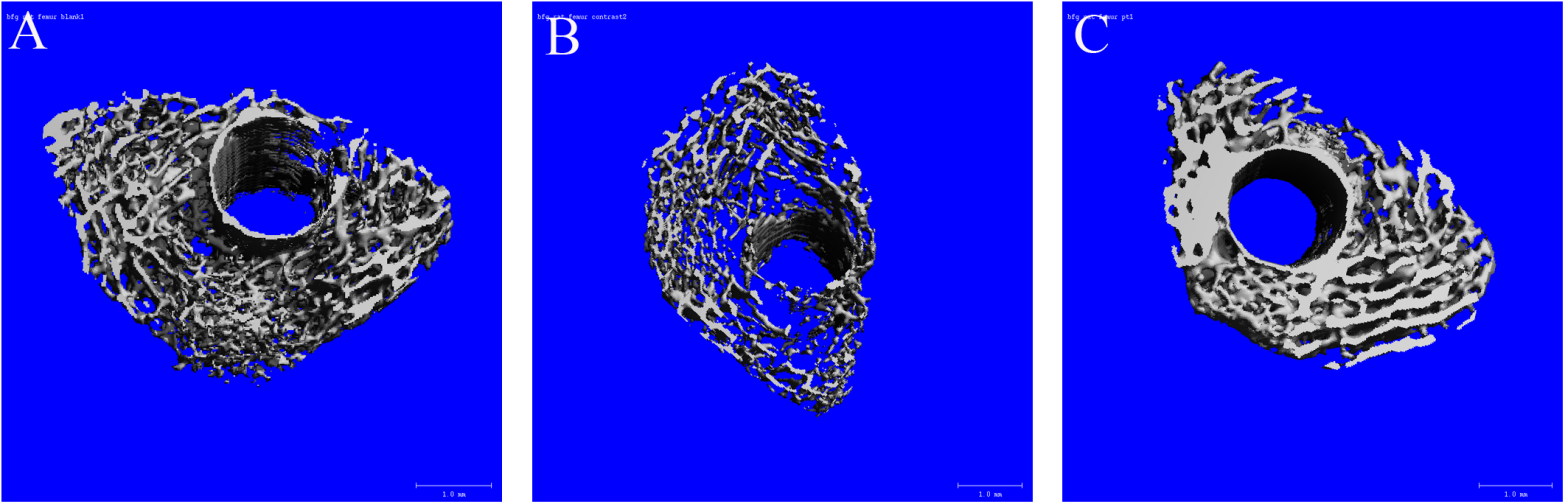
Representative 3D reconstruction images of the trabecular bone around the implant from micro-CT. Blank group (**A**), control group (**B**) and PTH group (**C**). We can easily observe an obvious decrease in bone mass in the control group (**B**) and an obvious increase in the PTH group (**C**).

We easily observed differences between groups through the 3D reconstructions of trabecular bone around the implant, which corresponded to the quantitative findings (Fig 5A–5C).

### Interfacial fixation strength evaluation

The biomechanical push-out test was performed successfully with all specimens (n = 6 per group). The average strength to failure in the PTH group (139.82 ± 25.55 N) was significantly greater than that in the control group (49.48 ± 21.22 N, *p* < 0.01) and the blank group (101.75 ± 12.60 N, *p* < 0.05). We also observed a dramatic decrease in maximal fixation strength in the control group compared to the blank group (*p* < 0.01, Fig 2D).

### SEM evaluation

**Fig 6:**
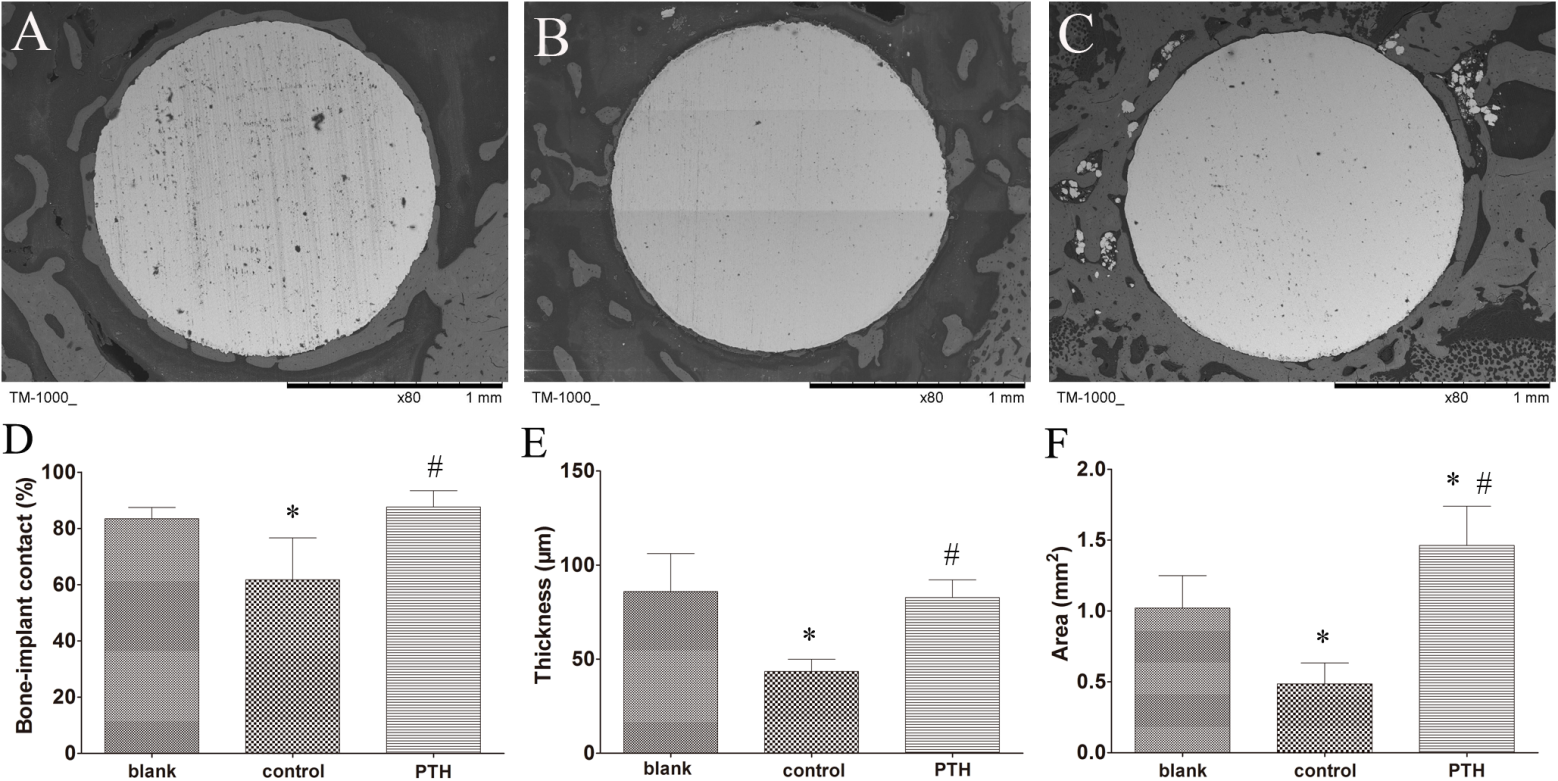
High-resolution SEM images of the bone–implant interface and the surrounding tissue. Representative SEM images from the blank group (**A**), control group, (**B**) and PTH group (**C**) at 80× magnification. SEM evaluations of bone–implant contact (**D**), thickness of lamellar bone around the implant (**E**) and trabecular bone area surrounding the implant (**F**) in the three groups. *p < 0.05 vs. the blank group; #*p* < 0.05 vs. the control group.

SEM images revealed concentric bone formation around the surface for most implants in the blank and PTH groups (Fig 6A and 6C). However, the bone tissue around the implants was fragmented at the interface, and direct bone–implant integration was hardly observed in the control group (Fig 6B).

Evaluations of bone–implant contact indicated a 26.02% decrease following treatment with Ti particles in the control group (61.75 ± 14.87%) compared to that in the blank group (83.47 ± 3.99%; *p* = 0.005). While the contact was significantly increased following the administration of PTH[1–34] for 6 weeks in the PTH group (87 ± 5.81%) compared to that in the control group (*p* = 0.001), there were no significant differences between the blank group and PTH group (*p* = 0.753; Fig 6D). The lamellar bone around the implant was much thicker in the blank group (85.90 ± 20.21 μm) and PTH group (82.72 ± 9.44 μm) relative to that in the control group (43.46 ± 6.44 μm; *p* = 0.000 and 0.001, respectively). There were no significant differences between the blank group and PTH group (*p* = 0.920; Fig 6E). A larger trabecular bone area was also observed in the PTH group (1.46 ± 0.00 mm^2^) relative to that in both the blank group (1.02 ± 0.00 mm^2^, *p* = 0.014) and control group (0.49 ± 0.00 mm^2^, *p* = 0.000). A significant decrease in trabecular bone area in the control group was observed following treatment with Ti particles compared to the blank group (*p* = 0.014; Fig 6F).

### Mineral apposition rate evaluation

**Fig 7:**
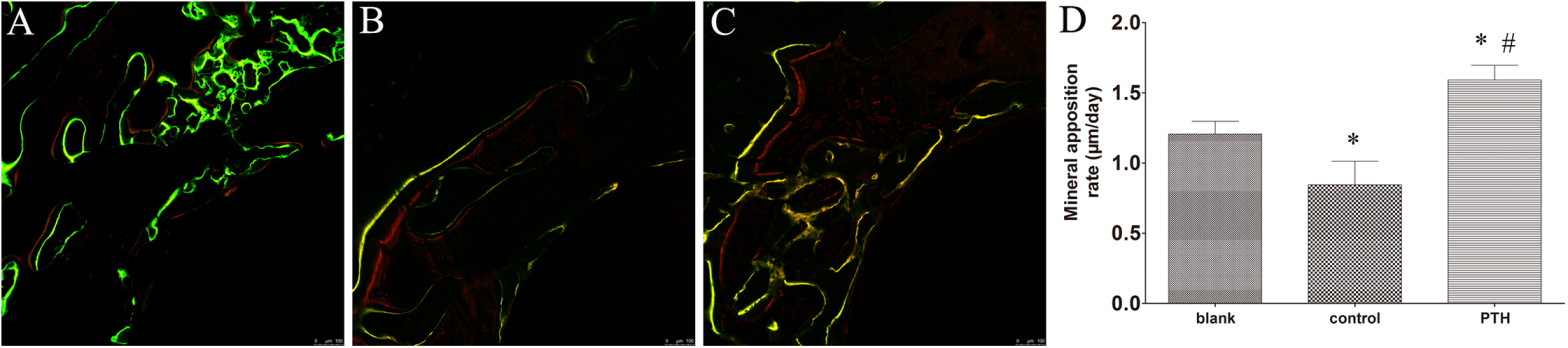
Periprosthetic bone was labeled during bone regeneration and remodeling by calcein (green) and tetracycline hydrochloride (red). Representative labeling images from the blank group (**A**), control group (**B**) and PTH group (**C**). Mineral apposition rate in the three groups. *p < 0.05 vs. the blank group; #*p* < 0.05 vs. the control group.

The mineral apposition rate of periprosthetic bone was detected by the distance between the parallel fluorescent bands. Six weeks postoperatively, the two fluorescent labels (green bands, calcein; red bands, tetracycline hydrochloride) were clear. No mineral apposition at the implant–bone interface was observed in the control group. In addition, the mineral apposition rate was measured only in the area of peripheral reconstruction (Fig 7A–7C). According to statistical analysis, there was a significant difference in the mineral apposition rate between the blank group (1.21 ± 0.09 μm/day) and control group (0.85 ± 0.17 μm/day, *p* = 0.001). Moreover, they were all substantially lower than in the PTH group (1.59 ± 0.10 μm/day, *p* = 0.000; Fig 7D).

### TRAP staining

**Fig 8:**
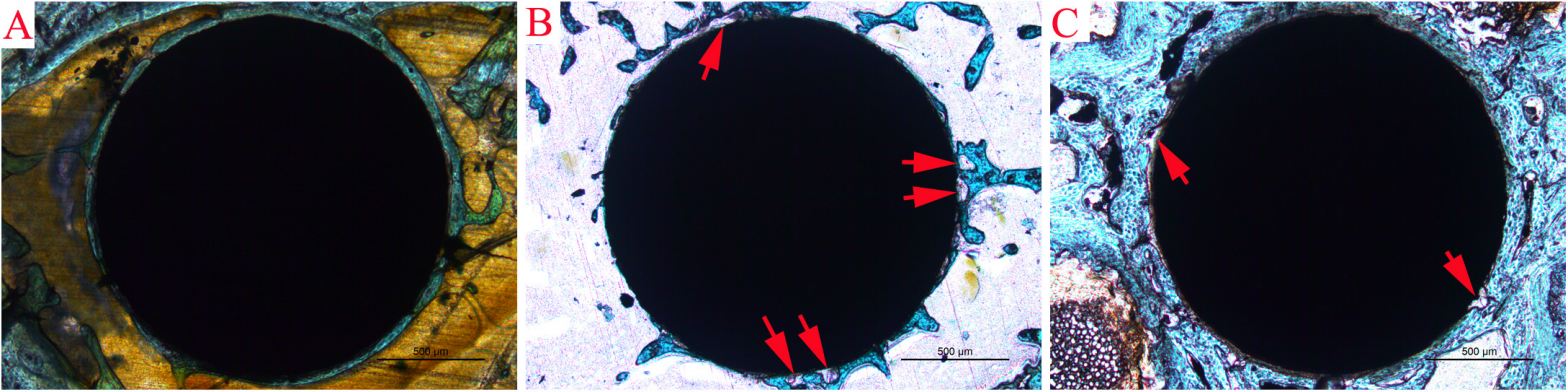
TRAP staining images from the three groups. (**A**) Blank group, well-formed bone–implant integration with trabecular bone surrounding the implant; (**B**) control group, obvious osteolysis and local trabecular bone disruption (red arrows) were observed at the bone–implant interface; and (**C**) improved bone–implant integration with thicker trabecular bone and reduced trabecular bone disruption (red arrows) were observed.

TRAP staining revealed the same bone morphogenesis around the implants as that following SEM. In the blank group, well-formed bone–implant integration with trabecular bone surrounding the implant was observed (Fig 8A). In the control group, obvious osteolysis occurred around the implant, trabecular bone was irregularly rare, and direct bone–implant integration was hardly observed. Local trabecular bone disruption and fibrous tissue membrane were also observed at the bone–implant interface (Fig 8B). In the PTH group, improved bone–implant integration with thicker trabecular bone and reduced trabecular bone disruption were observed compared to the control group (Fig 8C).

## Discussion

Our data support the hypothesis that intermittent administration of PTH[1–34] can prevent particle-induced osteolysis by enhancing periprosthetic bone formation in a rat model. These results provide radiological, biomechanical, and histological evidence to suggest that periprosthetic bone formation has been promoted. This study also demonstrated a potential therapeutic strategy using PTH[1–34] for periprosthetic osteolysis.

Intra-articular injections of particles (polyethylene particles or Ti particles, for example) imitated implant wear and germinated the process of periprosthetic osteolysis [38, 39]. Animals that received Ti particles in the control group exhibited obvious bone loss with lower BMD and significantly lower fixation strength than both the blank and PTH groups. These results demonstrate that intra-articular injection of Ti particles results in periprosthetic osteolysis, which is similar to aseptic loosening in the clinical setting. The rat model of periprosthetic osteolysis is a simple and reproducible system that is suitable for studying the effects of particle-induced bone loss [3, 38]. In our study, some procedures were carried out to minimize the effects of surgery on the results. Adherent endotoxins on Ti particles were removed to exclude their effects on osteoclastogenesis and the production of IL–1β, IL–6, and TNF-α [40]. The bone tunnel was created by a low-speed surgical drill to avoid heat-induced bone necrosis at the implant–bone interface. In addition, the following potential side effects of Ti particles were considered: metal ions released into the tissue surrounding implants, body fluid (serum and urine), and distant organs (lymph nodes, spleen, and liver) [41]; induction of proliferation; and differentiation of T-lymphocytes to produce excessive cytokines including IL–1, IL–6 and TNF-α, which would activate a series of systemic immune responses. We did not observe any major side effects in the rats.

PTH[1–34] enhances bone formation with intermittent and short-term administration in animals and humans [28, 42] and induces a significant increase in alkaline phosphatase (ALP) in serum and calcium concentrations with high-level dosages [43]. Intermittent administration of PTH[1–34] simultaneously promotes bone formation and resorption, but has a greater effect on bone formation than resorption, leading to net bone gain [44]. Until now, only a few studies have evaluated the efficacy of PTH[1–34] on periprosthetic osteolysis, although many studies have focused on its positive effects on the osteointegration of implants in cancellous bone [35, 36, 45]. PTH[1–34] has been shown to improve osteointegration of implants that are press-fit inserted into cancellous bone [35]. Intermittent administration of PTH[1–34] promotes periprosthetic cancellous bone formation, which is an anabolic factor as the mechanical loading [36]. In our study, even with a small sample size and evaluation only 6 weeks after surgery, increases in BMD and BV/TV and improvement in push-out strength were significantly correlated. PTH[1–34] has been proven to increase BMD, Tb.Th, Tb.N, and connectivity, thus promoting bone strength and reducing the rick of fracture in animals and patients [28, 46–48]. A previous study used micro-CT to evaluate bone microarchitecture, and demonstrated that the volume and thickness of cortical and cancellous bone were increased following the administration of PTH[1–34] [49]. What we found regarding the effects of PTH[1–34] on BMD, BV/TV, Tb.Th, and Tb.Sp of the periprosthetic bone was similar to that previous study.

We found that PTH[1–34], when administrated contemporaneously with the injection of Ti particles, completely abrogated the effects of the particles and prevented periprosthetic osteolysis. These results provide tissue-level insight into the mechanisms of the effects of Ti particles on implant fixation and how PTH[1–34] treatment prevents periprosthetic osteolysis. Bone mineral apposition rates were depressed following particle injection, revealing that the activity of osteoblasts in peri-implant trabecular bone was inhibited. PTH[1–34] significantly increased the mineral apposition rate in the PTH group, indicating an increase in osteoblast vigor or osteoblast number, or both. These findings are coincident with current knowledge regarding the effects of particles on osteoblasts both *in vitro* and *in vivo* [50, 51].

The dosages of PTH[1–34] administrated in previous studies have varied, depending on the experiment. The effective dosage of PTH[1–34] bone anabolic treatment ranges between 10 and 80 μg/kg/day in animal models with low-density bone [52–59]. A previous study found that, at dosages between 5 μg/kg/day and 75 μg/kg/day, PTH[1–34] stimulated morphometric and biomechanical parameters in a dose-dependent manner [45]. The low dose (5 μg/kg/day) had a minimal effect, which was not always statistically significant, while the high doses (25 and 75 μg/kg/day) induced stronger effects. PTH[1–34] enhances the bone mineralization apposition rate at the spinal fusion site in a dose-dependent manner, and low-dose (4 μg/kg/day) administration over a longer duration might also be beneficial [60]. Therefore, the high-dose administration with a short duration that we used in our study would be beneficial. The dosage of PTH[1–34] at 60 μg/kg/day was chosen because it has been widely used and showed a substantial anabolic effect in rats in previous studies [33, 61–63]. However, 60 μg/kg/day is a suprapharmacological dose of the human equivalent in rats. Our study provides little insight to whether therapeutic levels of intermittent PTH could be used to treat particle-induced osteolysis.

One concern regarding the administration of PTH[1–34] for the treatment of periprosthetic osteolysis is that it may enhance the risk of osteosarcoma, a malignant tumor that mostly occurs in adolescents and growing young adults. Its morbidity is inferior to 0.2% of all diagnosed cancers annually in the U.S. [64]. A preclinical study found that Fisher 344 rats administered PTH for 2 years had a significantly increased percentage of developing osteosarcoma [65]. However, they found that it appeared safe at a low dose (5 μg/kg/day, 3.4-fold of human equivalent dose by Food and Drug Administration [FDA]); however, a high dose (30 μg/kg/day) for 20–24 months (70–80% of the rat lifespan) induced osteosarcoma in 20% of rats in a subsequent study [66]. Therefore, the treatment duration of PTH is limited to 2 years (2–3% of the human lifespan). In addition, the U.S. FDA issued a mandatory black-box warning that “teriparatide should not be prescribed for patients with an increased baseline risk for osteosarcoma.” However, only two possible osteosarcoma cases have been observed in half a million patients administered PTH [67], indicating that the risk may be negligible in humans. A 7-year cancer surveillance study in the U.S. found that all patients with primary osteosarcoma had no prior PTH treatment, and there was no causal association between osteosarcoma and teriparatide treatment [68], which further strengthened the hypothesis that osteosarcoma induced by PTH in rats may not correlate with humans. Moreover, no increased risks of bone tumor were observed in patients with primary or secondary hyperparathyroidism [69, 70].

There are some limitations associated with our study. First, a suprapharmacological dose was selected and the results provided little insight into whether therapeutic levels of intermittent PTH could be used to treat particle-induced osteolysis. Second, we used a growing rat model, which does not mimic adult joint replacement patients with periprosthetic osteolysis. Further studies are needed to establish a clinically relevant Ti particle-induced periprosthetic osteolysis model in skeletally mature rats and evaluate whether therapeutic levels of PTH[1–34] prevent bone loss by promoting bone formation. In addition, the results do not answer the question regarding whether PTH[1–34] would have similar effects if the administration had been delayed. It is possible that a lack of osteolysis in the PTH group, owing to bone formation around the implant which prevents the ingress of particles along the interface, removing a significant stimulus of bone resorption. Even so, the results provide evidence that early promotion of periprosthetic bone formation is an effective strategy to prevent particle-induced osteolysis.

Clinically, endosseous implantation is a common procedure in orthopedics; however, consequent bone loss around the implant always leads to a substantially poor prognosis. In our study, a prevention strategy was examined, because PTH[1–34] treatment was contemporaneous with the administration of Ti particles. The promotion of implant anchorage in the osteolysis model provides a promising approach to prevent periprosthetic osteolysis by PTH[1–34] administration.

## Conclusions

Administration of PTH[1–34] is effective for preventing particle-induced periprosthetic osteolysis though the promotion of periprosthetic bone formation.

## Supporting Information

S1 Fig Load-elongation curves and maximal fixation strength data of biomechanical test for specimens in the three groups. (PDF)

S2 Fig 3D reconstruction images of micro-CT scan of each specimen in the three groups. (PDF)

S3 Fig SEM images of each specimen in the three group and the data of bone-implant contact, trabecular bone thickness, and bone area in the visual field collected by the IPP software. (PDF)

S1 Table Data of bone mineral density of each specimen in the three groups. (PDF)

S2 Table Micro-CT evaluations of each specimen in the three groups. (PDF)

S3 Table Data of mineral apposition rate collected by the IPP software. (PDF)
