# Supplementary material for: Intermittent Administration of Parathyroid Hormone [1–34] Prevents Particle-Induced Periprosthetic Osteolysis in a Rat Model
Source: PLoS One. 2015 Oct 6;10(10):e0139793. doi: 10.1371/journal.pone.0139793 (PMC4595472; doi:10.1371/journal.pone.0139793)
Supplement: S1 Fig — (PDF) [file pone.0139793.s001.pdf]

Supporting data for figure 3.

Maximal fixation strength (N) from each specimen of the three groups.

Blank group

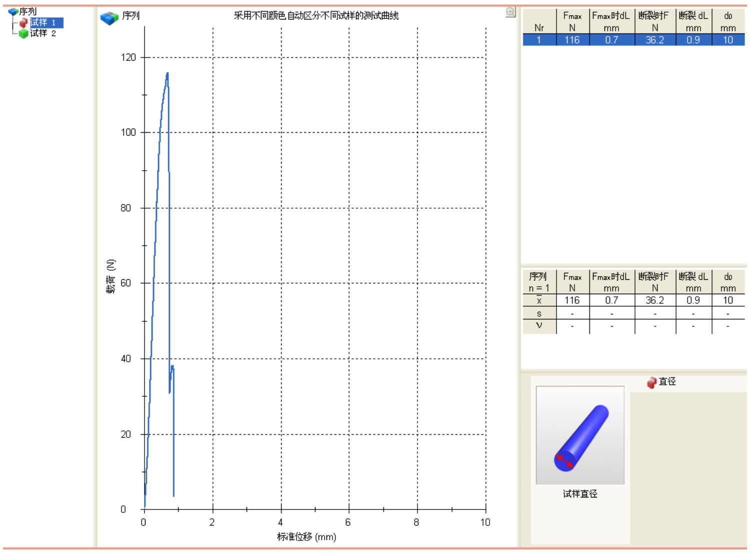

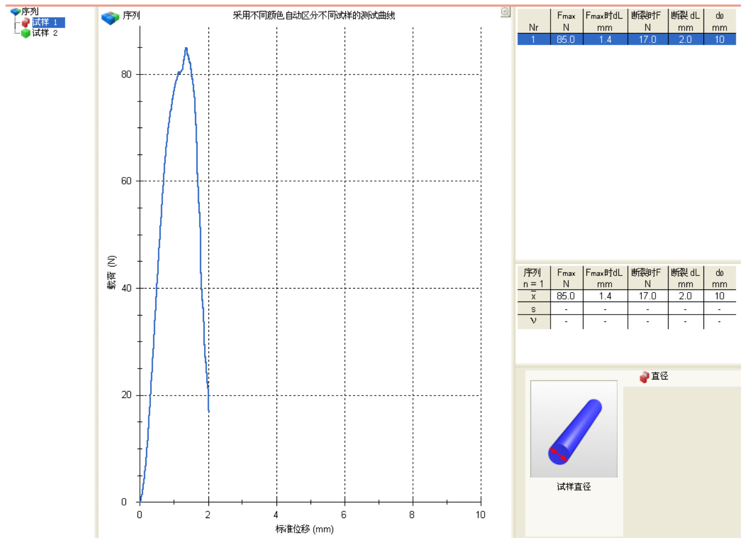

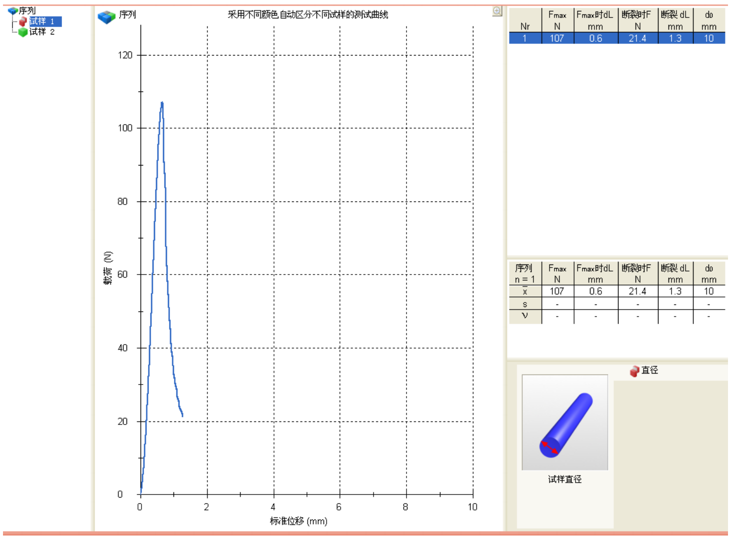

序列
试样 1
试样 2

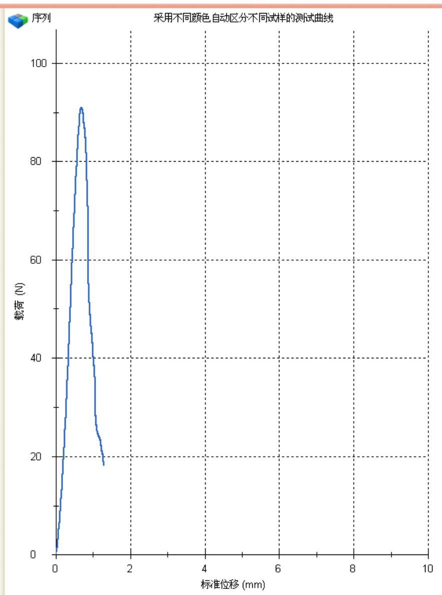

Nr	Fmax<br>N	Fmax时dL<br>mm	断裂时F<br>N	断裂 dL<br>mm	do<br>mm
1	91.1	0.7	18.2	1.3	10

序列<br>n = 1	Fmax<br>N	Fmax时dL<br>mm	断裂时F<br>N	断裂 dL<br>mm	do<br>mm
$\bar{x}$	91.1	0.7	18.2	1.3	10
s	-	-	-	-	-
ν	-	-	-	-	-

直径

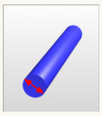

试样直径

序列
试样 1
试样 2

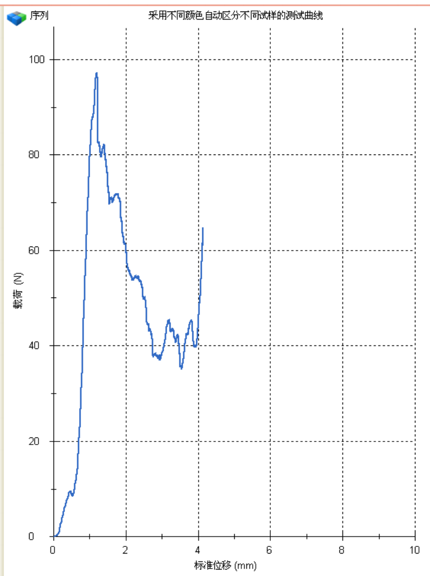

Nr	Fmax<br>N	Fmax时dL<br>mm	断裂时F<br>N	断裂 dL<br>mm	do<br>mm
1	97.4	1.2	-	-	10

序列<br>n = 1	Fmax<br>N	Fmax时dL<br>mm	断裂时F<br>N	断裂 dL<br>mm	do<br>mm
$\bar{x}$	97.4	1.2	-	-	10
s	-	-	-	-	-
ν	-	-	-	-	-

直径

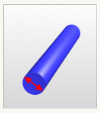

试样直径

序列
试样 1
试样 2

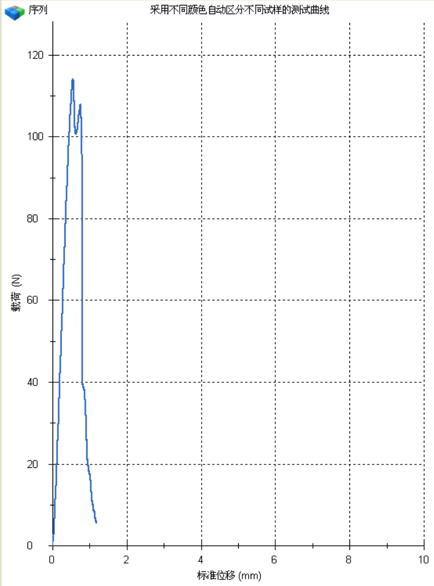

Nr	Fmax<br>N	Fmax时dL<br>mm	断裂时F<br>N	断裂 dL<br>mm	do<br>mm
1	114	0.5	5.70	1.2	10

序列<br>n = 1	Fmax<br>N	Fmax时dL<br>mm	断裂时F<br>N	断裂 dL<br>mm	do<br>mm
$\bar{x}$	114	0.5	5.70	1.2	10
s	-	-	-	-	-
ν	-	-	-	-	-

直径

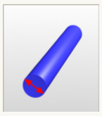

试样直径

Control group

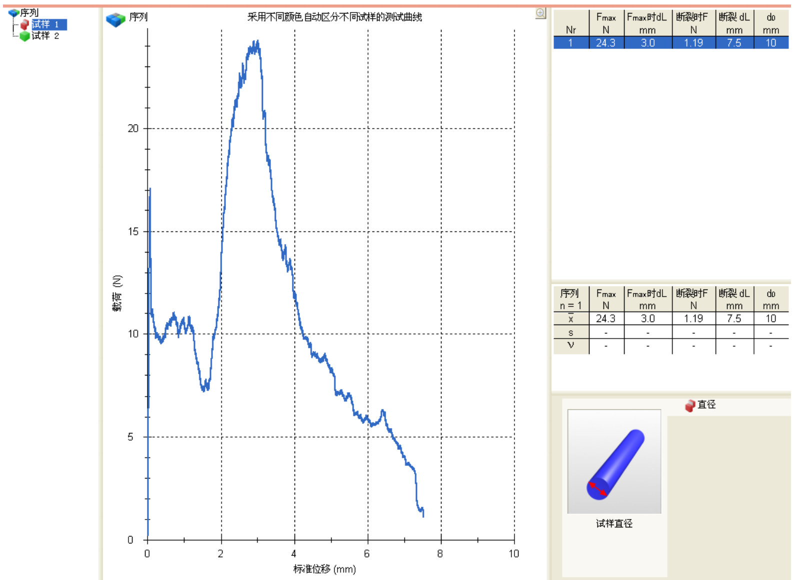

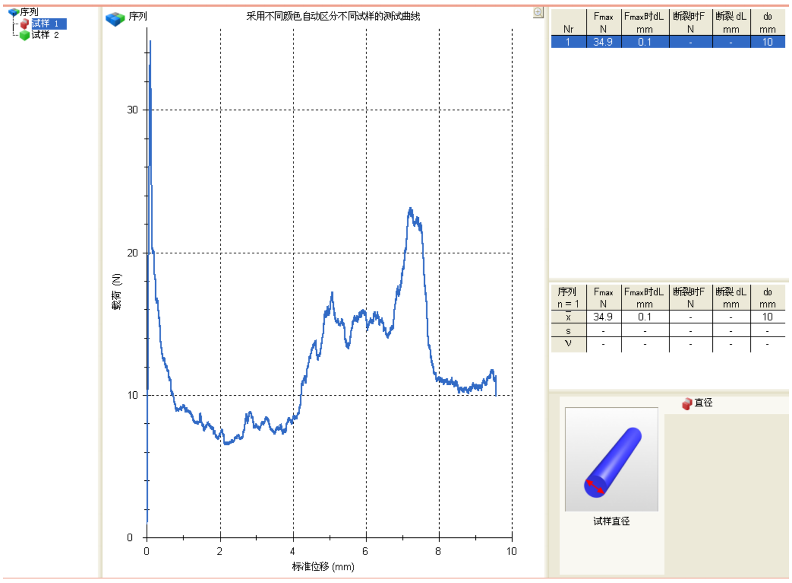

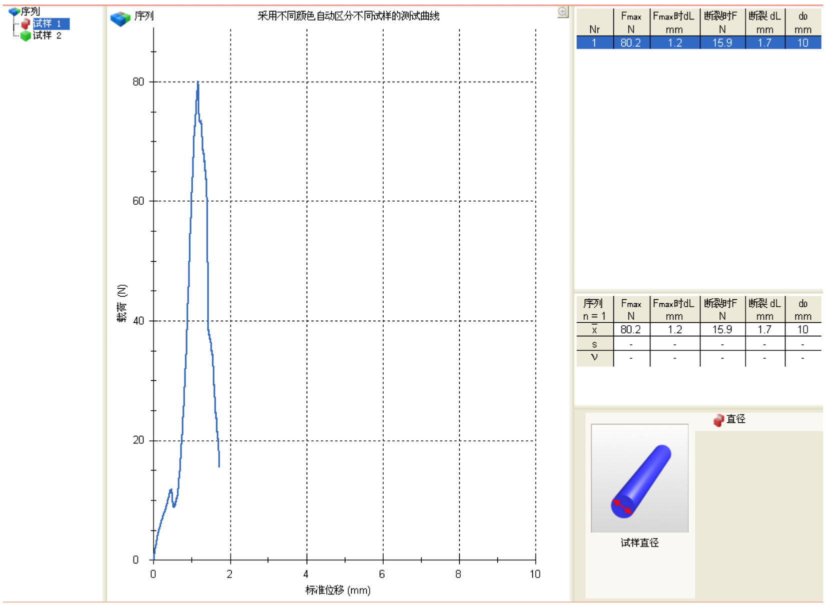

序列
试样 1
试样 2

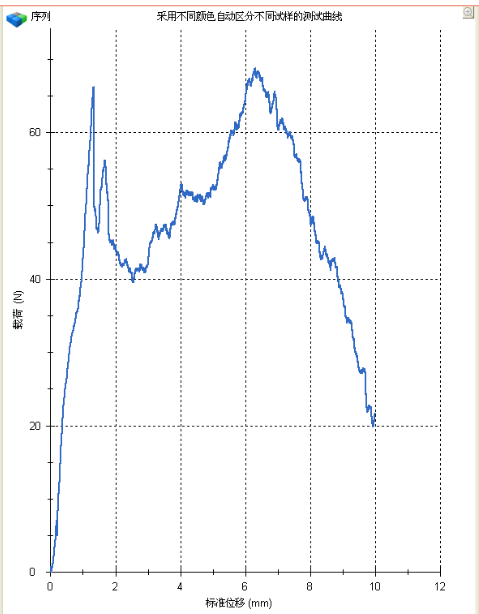

Nr	Fmax N	Fmax时dL mm	断裂时F N	断裂 dL mm	do mm
1	68.8	6.3	-	-	10

序列 n = 1	Fmax N	Fmax时dL mm	断裂时F N	断裂 dL mm	do mm
x̄	68.8	6.3	-	-	10
s	-	-	-	-	-
ν	-	-	-	-	-

直径

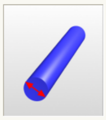

试样直径

序列
试样 1
试样 2

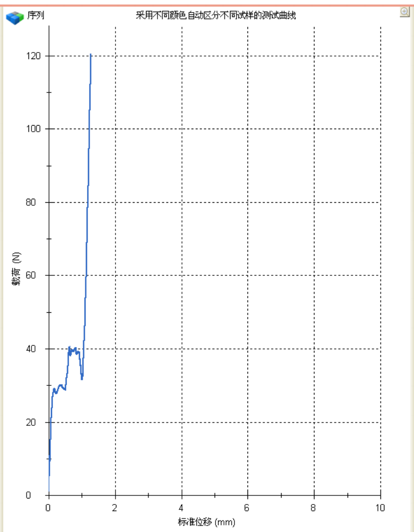

Nr	Fmax N	Fmax时dL mm	断裂时F N	断裂 dL mm	do mm	dL(1) %	标准位移(1) mm	F(1) N
1	121	1.3	-	-	10	0.79	0.80	40.43

序列 n = 1	Fmax N	Fmax时dL mm	断裂时F N	断裂 dL mm	do mm	dL(1) %	标准位移(1) mm	F(1) N
x̄	121	1.3	-	-	10	0.79	0.80	40.43
s	-	-	-	-	-	-	-	-
ν	-	-	-	-	-	-	-	-

直径 10

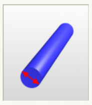

试样直径

序列
试样 1
试样 2

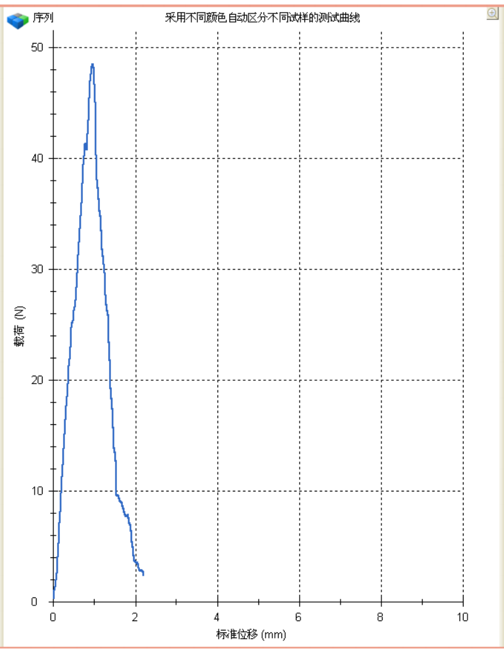

Nr	Fmax N	Fmax时dL mm	断裂时F N	断裂 dL mm	do mm
1	48.5	1.0	2.42	2.2	10

序列 n = 1	Fmax N	Fmax时dL mm	断裂时F N	断裂 dL mm	do mm
x̄	48.5	1.0	2.42	2.2	10
s	-	-	-	-	-
ν	-	-	-	-	-

直径

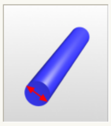

试样直径

PTH group

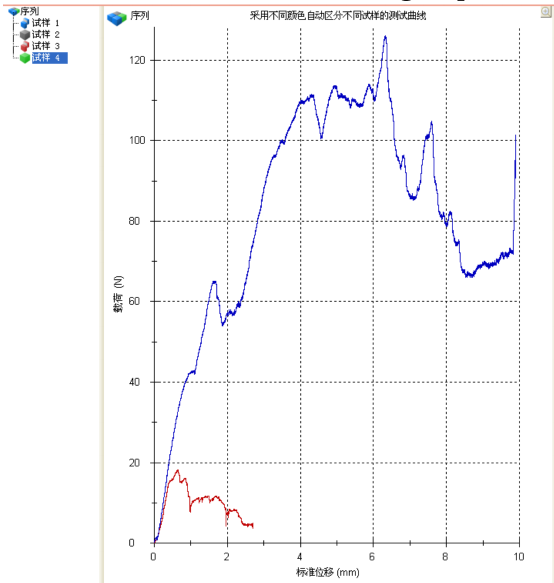

Nr	Fmax N	Fmax时dL mm	断裂时F N	断裂 dL mm	d0 mm
1	18.2	0.6	4.65	2.7	1.5
⊕2	-	-	-	-	1.5
3	126	6.3	-	-	1.5

序列 n = 2	Fmax N	Fmax时dL mm	断裂时F N	断裂 dL mm	d0 mm
x̄	72.2	3.5	4.65	2.7	1.5
s	76.3	4.0	-	-	0.000
ν	105.67	116.83	-	-	0.00

直径

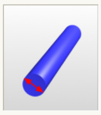

试样直径

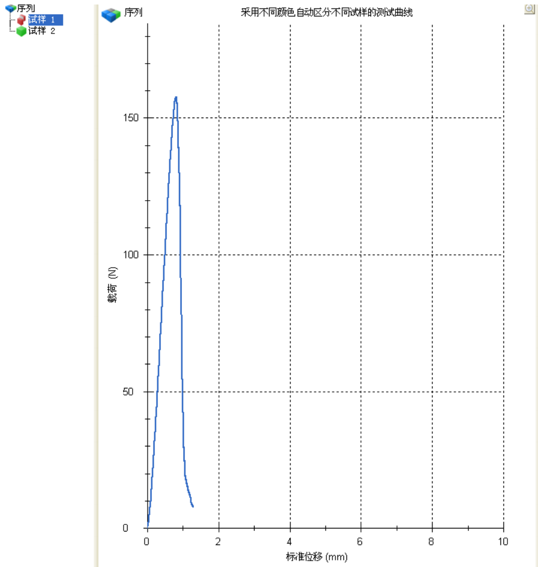

Nr	Fmax N	Fmax时dL mm	断裂时F N	断裂 dL mm	d0 mm
1	158	0.8	7.89	1.3	10

序列 n = 1	Fmax N	Fmax时dL mm	断裂时F N	断裂 dL mm	d0 mm
x̄	158	0.8	7.89	1.3	10
s	-	-	-	-	-
ν	-	-	-	-	-

直径

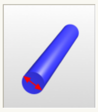

试样直径

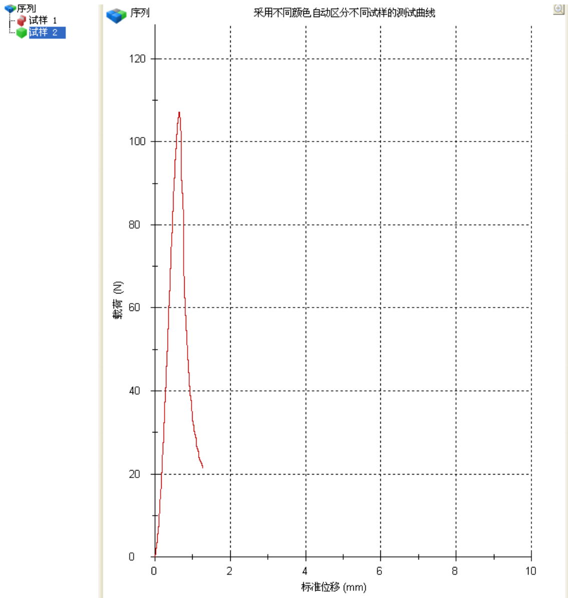

Nr	Fmax N	Fmax时dL mm	断裂时F N	断裂 dL mm	d0 mm
1	107	0.6	21.4	1.3	10

序列 n = 1	Fmax N	Fmax时dL mm	断裂时F N	断裂 dL mm	d0 mm
x̄	107	0.6	21.4	1.3	10
s	-	-	-	-	-
ν	-	-	-	-	-

直径

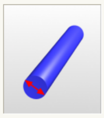

试样直径

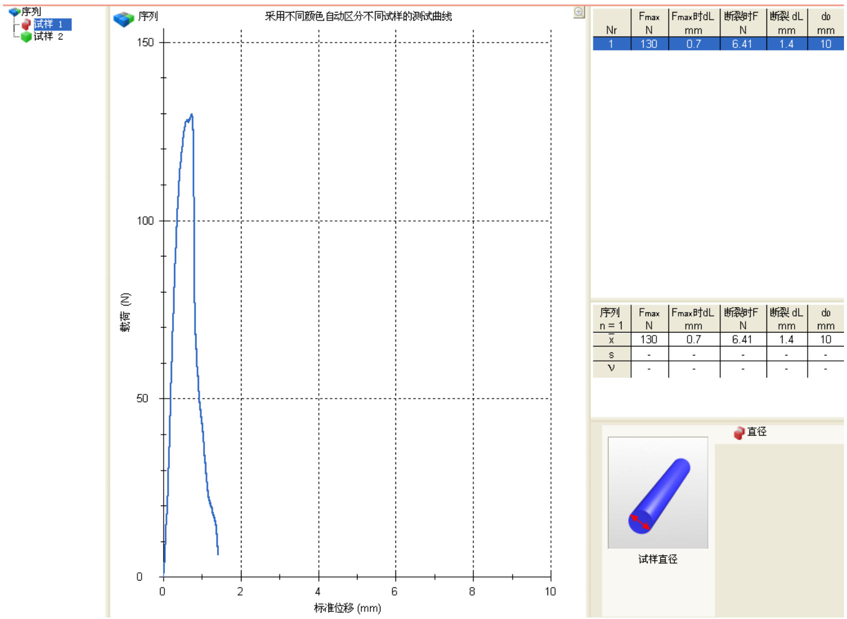
序列
试样 1
试样 2
序列
采用不同颜色自动区分不同试样的测试曲线
载荷 (N)
标准位移 (mm)
0
50
100
150
0
2
4
6
8
10
Nr | Fmax N | Fmax时dL mm | 断裂时F N | 断裂 dL mm | do mm
1 | 130 | 0.7 | 6.41 | 1.4 | 10
序列 n = 1 | Fmax N | Fmax时dL mm | 断裂时F N | 断裂 dL mm | do mm
x̄ | 130 | 0.7 | 6.41 | 1.4 | 10
s | - | - | - | - | -
ν | - | - | - | - | -
直径
试样直径

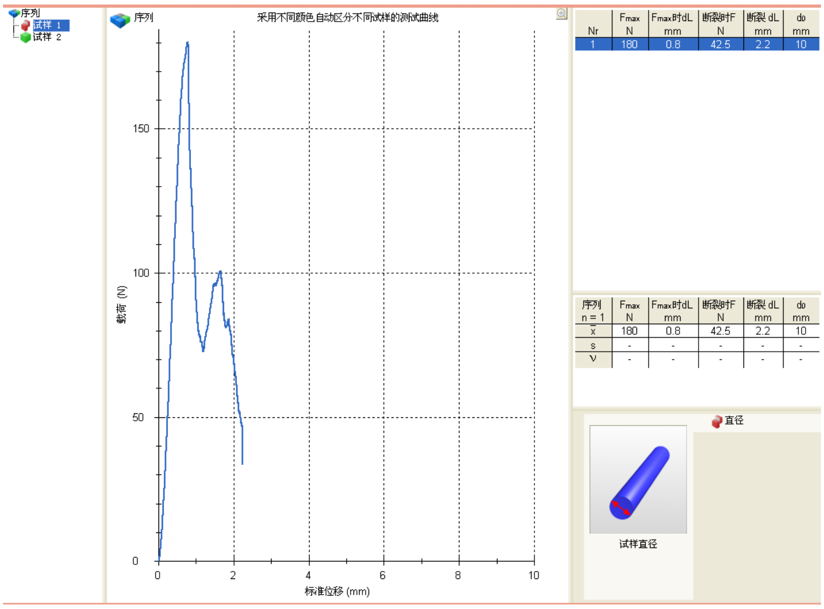
序列
试样 1
试样 2
序列
采用不同颜色自动区分不同试样的测试曲线
载荷 (N)
标准位移 (mm)
0
50
100
150
0
2
4
6
8
10
Nr | Fmax N | Fmax时dL mm | 断裂时F N | 断裂 dL mm | do mm
1 | 180 | 0.8 | 42.5 | 2.2 | 10
序列 n = 1 | Fmax N | Fmax时dL mm | 断裂时F N | 断裂 dL mm | do mm
x̄ | 180 | 0.8 | 42.5 | 2.2 | 10
s | - | - | - | - | -
ν | - | - | - | - | -
直径
试样直径

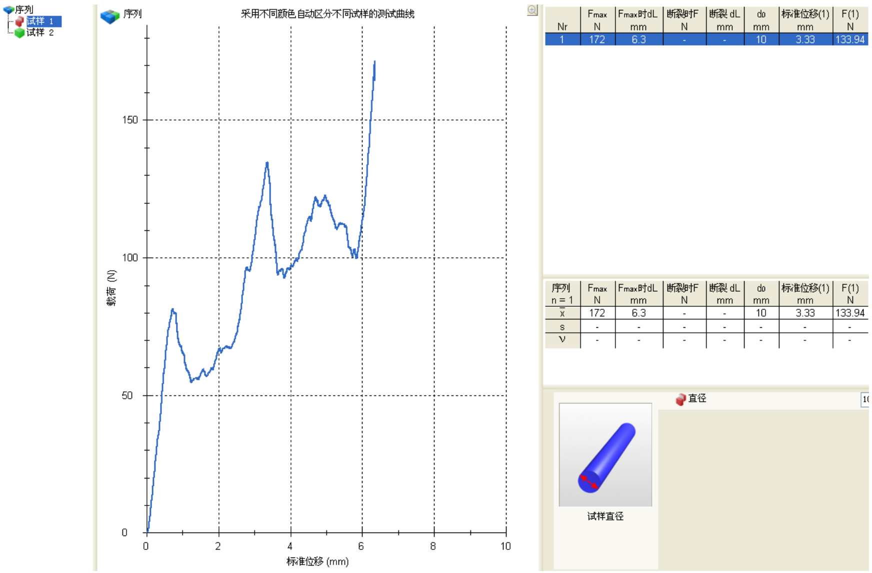
序列
试样 1
试样 2
序列
采用不同颜色自动区分不同试样的测试曲线
载荷 (N)
标准位移 (mm)
0
50
100
150
0
2
4
6
8
10
Nr | Fmax N | Fmax时dL mm | 断裂时F N | 断裂 dL mm | do mm | 标准位移(1) mm | F(1) N
1 | 172 | 6.3 | - | - | 10 | 3.33 | 133.94
序列 n = 1 | Fmax N | Fmax时dL mm | 断裂时F N | 断裂 dL mm | do mm | 标准位移(1) mm | F(1) N
x̄ | 172 | 6.3 | - | - | 10 | 3.33 | 133.94
s | - | - | - | - | - | - | -
ν | - | - | - | - | - | - | -
直径
试样直径

groups	Maximal fixation strength (N)					
blank	116	91.1	107	85	97.4	114
control	24.3	34.9	80.2	68.8	40.2	48.5
PTH	128.6	130	107	158	180	135.3
