# Supplementary material for: Intermittent Administration of Parathyroid Hormone [1–34] Prevents Particle-Induced Periprosthetic Osteolysis in a Rat Model
Source: PLoS One. 2015 Oct 6;10(10):e0139793. doi: 10.1371/journal.pone.0139793 (PMC4595472; doi:10.1371/journal.pone.0139793)

Supporting data for figure 5.

3D reconstruction images of each specimen in the three groups.

blank group

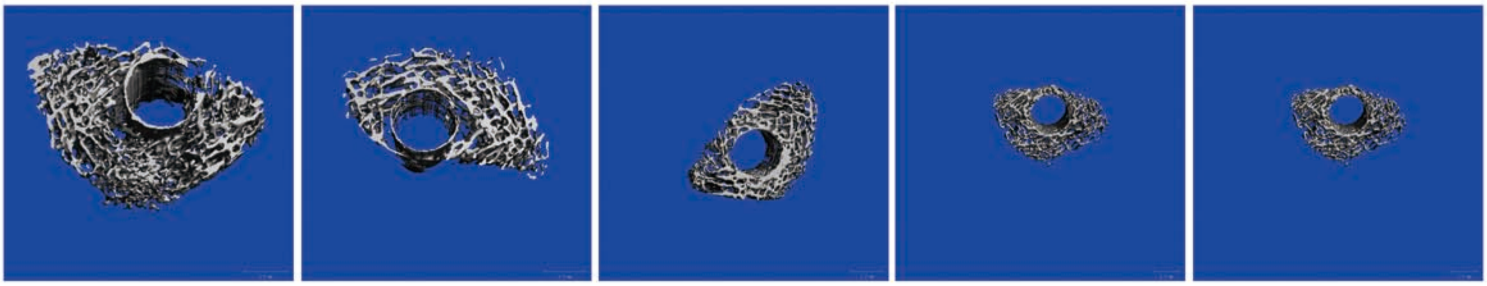

control group

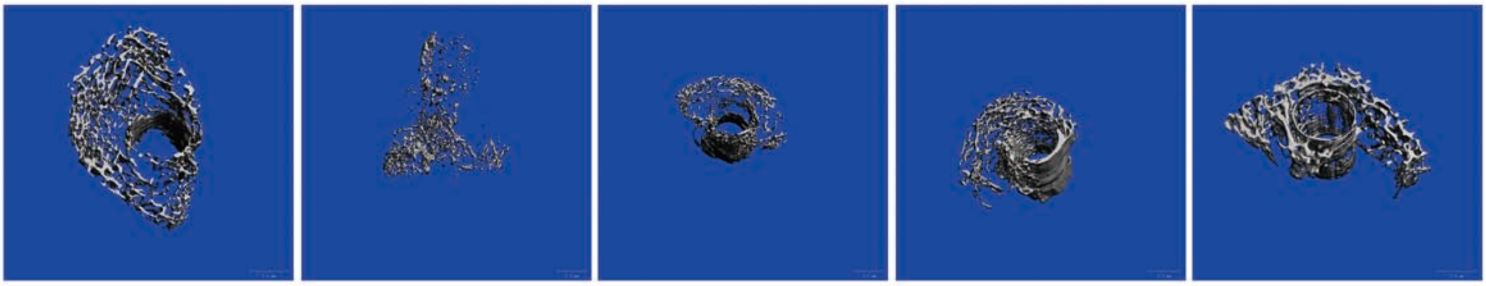

PTH group

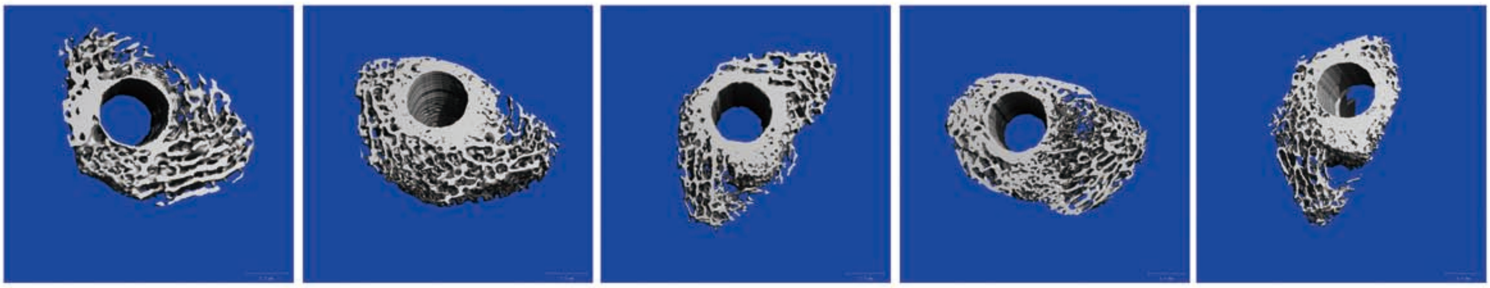

Supplement: S2 Fig — (PDF) [file pone.0139793.s002.pdf]
