# Supplementary material for: Intermittent Administration of Parathyroid Hormone [1–34] Prevents Particle-Induced Periprosthetic Osteolysis in a Rat Model
Source: PLoS One. 2015 Oct 6;10(10):e0139793. doi: 10.1371/journal.pone.0139793 (PMC4595472; doi:10.1371/journal.pone.0139793)
Supplement: S3 Fig — (PDF) [file pone.0139793.s003.pdf]

Supporting data for figure 6.

SEM images of each specimen in the three groups.

Blank group

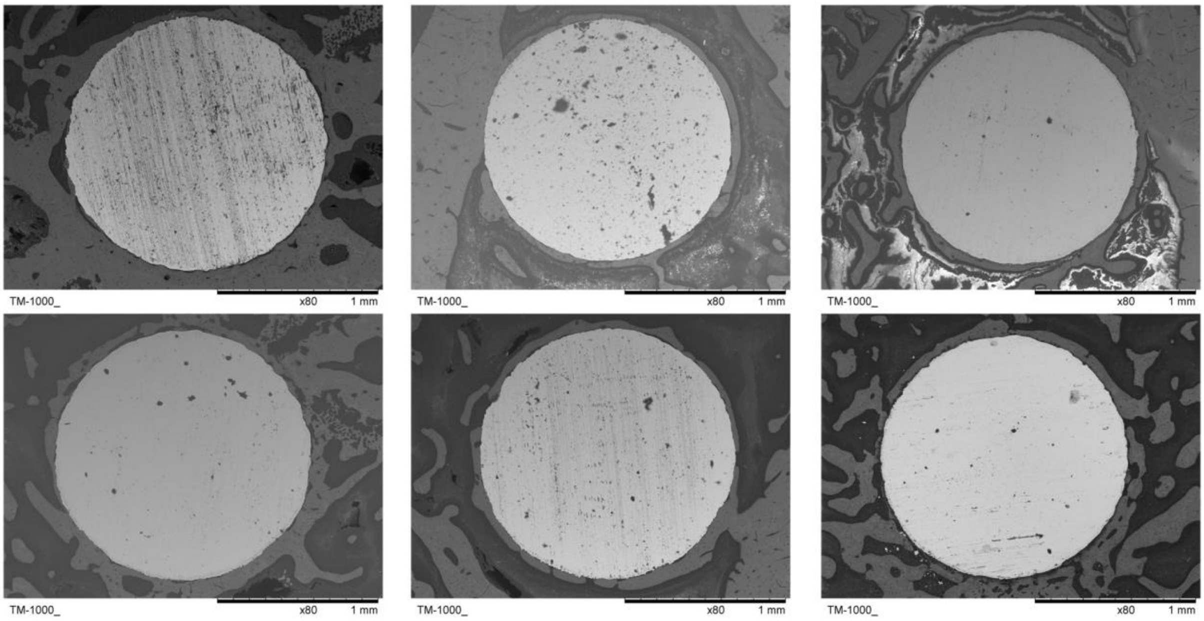

Control group

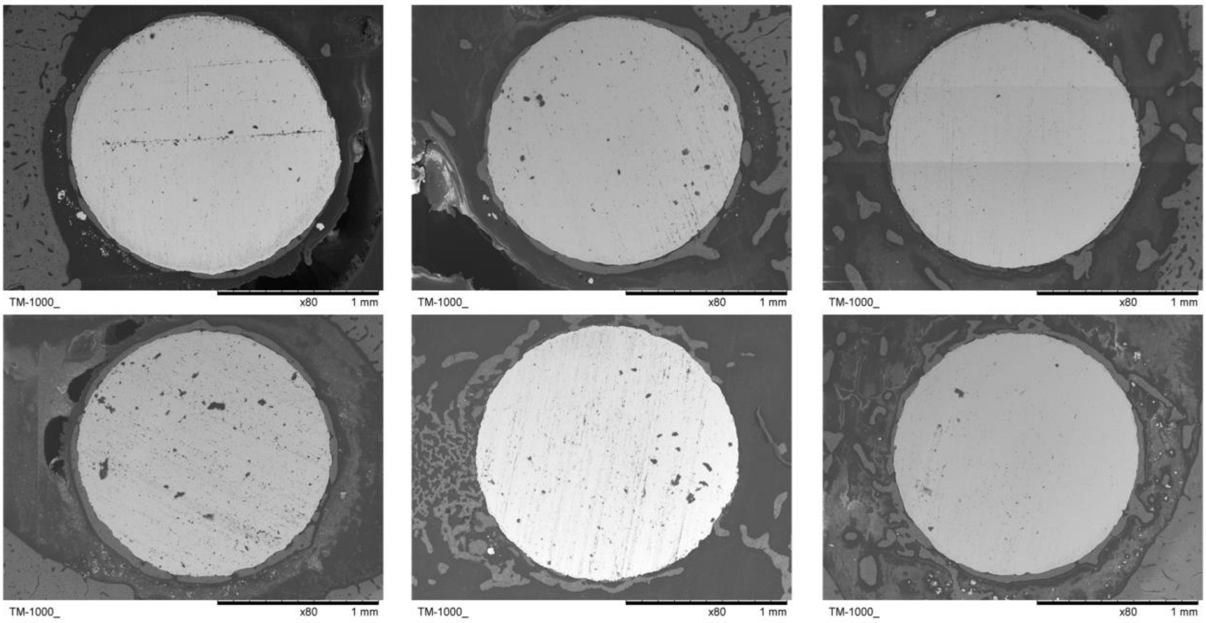

PTH group

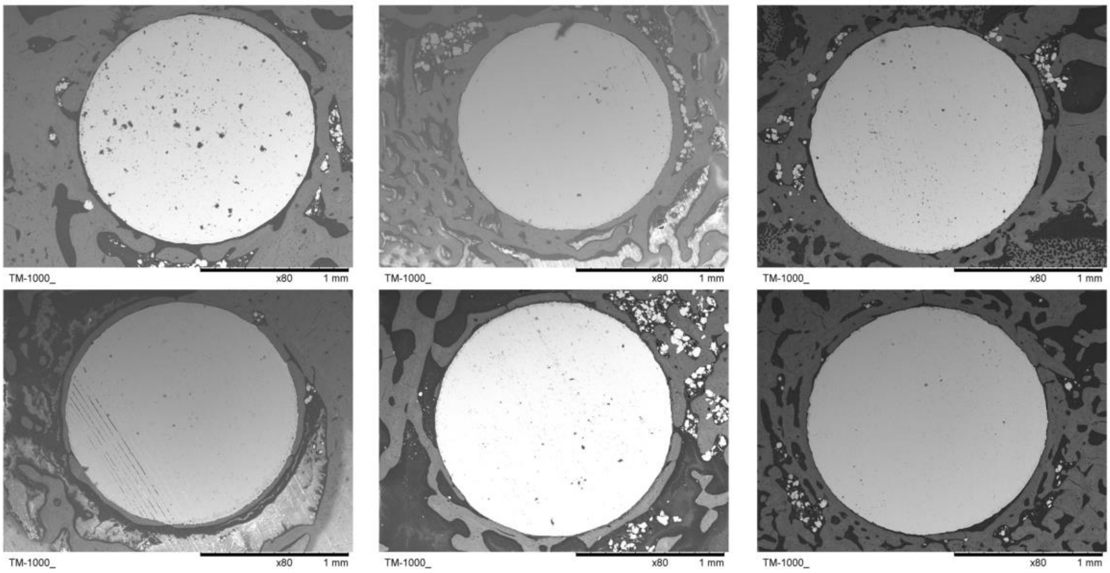

Data collected from IPP software.

groups		Bone-implant contact (%)	Trabecular bone thickness (μm)	Bone area (mm^2)
blank group	1	77.86	125.643	1.239042
	2	86.98	84.407	1.087797
	3	85.08	79.822	1.316846
	4	79.04	78.364	0.875996
	5	86.77	67.938	0.746034
	6	85.08	79.252	0.862145
Control group	1	66.06	48.228	0.764807
	2	73.54	47.581	0.474281
	3	39.32	35.21	0.352643
	4	69.56	48.272	0.456568
	5	47.16	46.295	0.382597
	6	74.84	35.196	0.487499
PTH group	1	82.14	90.17	1.610933
	2	94.05	85.622	1.771457
	3	83.82	76.922	1.428431
	4	95.9	69.637	1.577964
	5	84.73	78.653	0.961728
	6	85.18	95.369	1.423149
