# Supplementary material for: Intermittent Administration of Parathyroid Hormone [1–34] Prevents Particle-Induced Periprosthetic Osteolysis in a Rat Model
Source: PLoS One. 2015 Oct 6;10(10):e0139793. doi: 10.1371/journal.pone.0139793 (PMC4595472; doi:10.1371/journal.pone.0139793)
Supplement: S1 Table — (PDF) [file pone.0139793.s004.pdf]

Supporting data for figure 1.

Data of bone mineral density of each specimen in the three groups.

groups	bone mineral density (BMD, g/cm^2)					
Blank	0.159	0.171	0.161	0.178	0.17	0.167
Control	0.149	0.157	0.139	0.143	0.146	0.135
PTH	0.182	0.208	0.171	0.196	0.197	0.184
