# Supplementary material for: Intermittent Administration of Parathyroid Hormone [1–34] Prevents Particle-Induced Periprosthetic Osteolysis in a Rat Model
Source: PLoS One. 2015 Oct 6;10(10):e0139793. doi: 10.1371/journal.pone.0139793 (PMC4595472; doi:10.1371/journal.pone.0139793)
Supplement: S2 Table — (PDF) [file pone.0139793.s005.pdf]

Supporting data for table 1.

Micro-CT evaluations of each specimen in the three groups.

groups		BV/TV (%)	BS/TV (1/mm)	Tb.N (1/mm)	Tb.Th (μm)	Tb.Sp (μm)	connective density ($1/mm^3$)	structure model index
blank group	1	0.1391	39.0421	2.7938	0.0804	0.3558	28.3762	2.0874
	2	0.134	35.5168	2.379	0.098	0.364	31.7648	2.0997
	3	0.087	36.179	2.3766	0.0694	0.42	23.3814	2.1593
	4	0.0819	38.9103	2.0564	0.0971	0.4349	25.5947	2.0333
	5	0.1525	36.1727	2.0099	0.071	0.4857	22.174	2.0538
	6	0.1519	37.9779	1.5553	0.0527	0.5903	19.2264	2.0066
control group	1	0.0186	46.2018	0.8455	0.0617	1.1904	18.9241	3.8761
	2	0.0148	48.3734	0.3586	0.0413	2.7474	15.3873	3.6792
	3	0.0688	35.4697	1.4031	0.0761	0.728	15.4428	4.01244
	4	0.0626	31.6383	0.9909	0.0632	0.946	16.2408	3.6561
	5	0.1117	31.2693	1.7115	0.0514	0.6283	14.9816	3.429
	6	0.1057	16.5242	1.2547	0.0563	0.676	17.3854	4.1616
PTH group	1	0.3025	28.4939	3.065	0.138	0.3202	39.2231	0.8634
	2	0.3702	20.5885	3.1626	0.2105	0.2191	40.9241	0.9963
	3	0.2424	27.296	2.9966	0.1041	0.3256	41.2592	1.2387
	4	0.2439	26.2803	3.2049	0.1008	0.2359	43.0937	1.376
	5	0.3175	23.1729	2.8869	0.1469	0.3583	42.0853	1.3778
	6	0.3233	20.4075	3.2992	0.121	0.2051	41.0258	1.0258
