# Supplementary material for: Intermittent Administration of Parathyroid Hormone [1–34] Prevents Particle-Induced Periprosthetic Osteolysis in a Rat Model
Source: PLoS One. 2015 Oct 6;10(10):e0139793. doi: 10.1371/journal.pone.0139793 (PMC4595472; doi:10.1371/journal.pone.0139793)
Supplement: S3 Table — (PDF) [file pone.0139793.s006.pdf]

Supporting data for figure 7.

Data of mineral apposition rate collected by IPP software.

groups		Mineral apposition rate (μm/day)
Blank group	1	1.11564
	2	1.14274
	3	1.12927
	4	1.31694
	5	1.31306
	6	1.22045
Control group	1	0.73533
	2	0.90342
	3	1.05452
	4	1.00301
	5	0.75483
	6	0.62639
PTH group	1	1.51507
	2	1.64424
	3	1.67193
	4	1.53846
	5	1.45671
	6	1.72893
